# The Functions and Mechanism of a New Oligopeptide BP9 from Avian Bursa on Antibody Responses, Immature B Cell, and Autophagy

**DOI:** 10.1155/2019/1574383

**Published:** 2019-01-06

**Authors:** Xiu Li Feng, Man Man Zong, Guang Fang Zhou, Yang Zheng, Yuan Nan Yu, Rui Bing Cao, Pu Yan Chen, Mei Yang

**Affiliations:** ^1^Key Laboratory of Animal Microbiology of China's Ministry of Agriculture, College of Veterinary Medicine, Nanjing Agricultural University, Nanjing 210095, China; ^2^Hospital of Nanjing Agricultural University, Nanjing 210095, China

## Abstract

The bursa of Fabricius is an acknowledged central humoral immune organ unique to birds, which is vital to B cell differentiation and antibody production. However, the function and mechanism of the biological active peptide isolated from bursa on B cell development and autophagy were less reported. In this study, we isolated a new oligopeptide with nine amino acids Leu-Met-Thr-Phe-Arg-Asn-Glu-Gly-Thr from avian bursa following RP-HPLC, MODIL-TOP-MS, and MS/MS, which was named after BP9. The results of immunization experiments showed that mice injected with 0.01 and 0.05 mg/mL BP9 plus JEV vaccine generated the significant increased antibody levels, compared to those injected with JEV vaccine only. The microarray analysis on the molecular basis of BP9-treated immature B cell showed that vast genes were involved in various immune-related biological processes in BP9-treated WEHI-231 cells, among which the regulation of cytokine production and T cell activation were both major immune-related processes in WEHI-231 cells with BP9 treatment following network analysis. Also, the differentially regulated genes were found to be involved in four significantly enriched pathways in BP9-treated WEHI-231 cells. Finally, we proved that BP9 induced the autophagy formation, regulated the gene and protein expressions related to autophagy in immature B cell, and stimulated AMPK-ULK1 phosphorylation expression. These results suggested that BP9 might be a strong bursal-derived active peptide on antibody response, B cell differentiation, and autophagy in immature B cells, which provided the linking among humoral immunity, B cell differentiation, and autophagy and offered the important reference for the effective immunotherapeutic strategies and immune improvement.

## 1. Introduction

The bursa of Fabricius (BF) of chicken is a foundational model for immunology research, which provides some valuable insights into the central humoral immune function for human and mammal. The discovery and identification of the lymphatic system have a long and fascinating history [1], which emerged two major immune systems, namely, the cellular immune system represented by thymus and humoral immune system represented by the bursa of Fabricius (BF) [2, 3]. BF has made a far-reaching influence on two lineages of immune cells and becomes the basis for vaccination, cancer therapy, and drug development [4].

BF is a primary lymphoid organ for B cell development and gut-associated lymphoid tissue unique to the avian species [5]. IgM(+)IgG(+) B cells are the early present in BF, which are generated by Ag-dependent binding of MIgG to IgM(+) B cells in BF after hatching [6], which might be induced for further B cell differentiation by antigen-dependent attachment of maternal IgG in the medulla [7]. B cell differentiation and immunoglobulin diversification were accompanying with regulation of biological active molecular and activation of immune induction [8]. Bursin tripeptide (Lys-His-Gly-NH2) is reported to be the first B cell-differentiating hormone derived from BF [9, 10], selectively induces avian B cell differentiation [10], and promotes Ig switching from IgM to IgG [11]. Bursin-like peptide could significantly induce the strong immune response in mice immunized with the Japanese encephalitis virus (JEV) subunit vaccine [12]. Furthermore, bursal peptide BP8 could promote colony-forming pre-B formation and regulate B cell development [13], and BP5 regulated B cell development by promoting antioxidant defense [14]. Bursal pentapeptide-II (BPP-II) and BP5 regulated various pathways and immune-related biological processes in hybridoma cells secreting monoclonal antibody especial to JEV [15, 16]. Additionally, bursal pentapeptide-I (BPP-I) inhibited tumor cell proliferation and induced p53 expression [17]. B cell differentiation and development are the complex biological processes, including various gene expressions, gene regulation, and signal activation. Investigation of the immune induction of bursal-derived peptide had primarily been conducted following mouse immunization and immature B cell model, whereas little was known about the molecular basis of bursal peptides on immature B cell development and autophagy.

In this paper, we isolated a new oligopeptide BP9 with nine amino acids from BF and examined the inducing function of BP9 on antibody responses to JEV. Furthermore, we analyzed the gene expression profile and immune-related biological process network of WEHI-231 immature B cells after BP9 treatment and found that autophagy is one of important biological pathways for BP9-treated immature B cell line. These results provided some novel insights on the potential mechanism of bursal-derived peptides on humoral immune activation and B cell development and offered the important reference for the effective immunotherapeutic strategies and immune improvement.

## 2. Materials and Methods

### 2.1. Mice and WEHI-231 Cell Line

BALB/c female mice (6–8 weeks old, about 20 g) were obtained from Yangzhou University (Yangzhou, China). All of the animal experimental procedures were performed in accordance with the institutional ethical guidelines for animal experiments. WEHI-231 B cell lines, classified as immature B cells [18], were maintained in RPMI 1640 medium (Gibco) supplemented with 10% fetal bovine serum (FBS) (HyClone, USA), 1250 U/mL penicillin G, 0.5 mg/mL streptomycin sulfate, and 50 *μ*M 2-mercaptoethanol (Sigma, USA) in a 5% CO_2_ humidified incubator.

### 2.2. Reversed-Phase HPLC and Mass Analysis

The bursa extracts of BF were sequentially separated and purified as described for soluble polypeptide from the bursal sample as described in the previous reported method [16, 17] with some modification. Briefly, the crude extracts of BF from one-month-old avian were obtained by homogenization and centrifugation at 12,000 rpm for 45 min and then ultrafiltered with molecular weight less than 1 kDa at 4°C for 48 h. The obtained sample was purified on a SinoChrom ODS-BP RP-HPLC affinity column (250 × 4.6 mm, Elite) using a linear gradient of acetonitrile (2–100%) with a DAD UV-V detector at 220 nm. RP-HPLC profile of peak at 11.19 min of the acetonitrile soluble fraction based on the elution time was collected and analyzed using matrix-assisted laser desorption ionization time-of-flight mass spectrometry (MALDI-TOF-MS) (Bruker) and then analyzed using MS/MS (Bruker) to obtain the exact amino acid sequence of bursa oligopeptide.

### 2.3. Peptide Synthesis

The bursal-derived peptide BP9 was synthesized by Nanjing GenScript Bioscience Co. Ltd. (China), with the purity over 97.8%, and was analyzed and confirmed by HPLC and mass spectrometry. Also, the irrelevant peptide (RMYEE) was synthesized to be used as peptide control, which was one peptide derived from BF, and did not produce the significant immune inducing roles on vaccination [19].

### 2.4. Mouse Immunization

The 4–6-week-old female BALC/c mice were randomly divided into five groups at 10 mice per group. JEV (NJ2008 strain) was conserved in our laboratory and was inactivated with 10^7^ pfu/0.1 mL. Three dosages of 0.01, 0.05, and 0.25 mg/mL BP9 were fully mixed with JEV antigen and then were fully emulsified with oil adjuvant, which did not affect the quality and the stability of the emulsion vaccine, and in this paper, we designed the water and only oil adjuvant as control to verified that the emulsified vaccines with BP9 were dependent of BP9 sequence. All the experimental vaccines were intraperitoneally injected into mice at 0.2 mL per mouse. Additionally, mice were immunized with three dosages of BP9 without JEV vaccine as the only peptide control, mice immunized with JEV vaccine and the irrelevant peptide (RMYEE, 0.05 mg/mL) were used as the peptide control, and mice immunized with JEV vaccine were used as the JEV vaccine control. All mice were thrice immunized at once every two weeks.

### 2.5. Antibody Detection

The samples of sera were collected from all the immunized mice before immunization and at six weeks after immunization to detect the antibody levels and antibody subtype especial to JEV with the ELISA method [16]. Simply, ELISA plate was coated with 2 *μ*g/mL JEV antigen diluted with 0.05 M (pH 9.6) carbonate buffer. After blocked and incubated with the collected serum samples, the ELISA wells were fortified with HRP-labeled IgG, IgG1, and IgG2a, respectively, treated with TMB substrate chromogenic solution, then added with 2 M H_2_SO_4_ buffer, and were determined by an enzyme analyzer at OD 450 nm value.

Additionally, the serum samples were collected from all immunized groups at one week after the second immunization to detect the neutralizing titers specific to JEV antigen in the sera with the conventional plaque reduction neutralization assays [20].

### 2.6. FCM and MTT Assay

Furthermore, at 7^th^ day after the second immunization, the splenic lymphocytes were isolated from all immunized mice and were incubated for 30 min with PE-CY5, FITC, and PE-labeled CD3, CD4, and CD8 monoclonal antibodies, respectively. After erythrocytes were disrupted and washed, the splenic lymphocytes were analyzed for T cell subtypes by flow cytometry (BD, FACSCalibur) [16].

Also, the isolated mouse splenic lymphocytes were seeded in a 96-well plate and treated with PBS, 10 ng/mL LPS, and 10 ng/mL Con A for 44 h, respectively, and the viability was determined with the MTT reagent (Sigma) [21].

### 2.7. WEHI-231 Cell Treatment

WEHI-231 cells were treated with BP9 from 0.001 to 50 *μ*g/mL for 44 h, inoculated with 10 *μ*L MTT (0.5 mg/mL) per well for 4 h, then added with 100 *μ*L DMSO per well, and read by an enzyme analyzer at OD 570 nm value to analyze the viability, as the following equation: the relative survival stimulate index (%) = ((absorbance of BP9 treatment − blank)/(absorbance of control − blank)) × 100%. Also, WEHI-231 cells were stimulated by BP9 at the experimental dosages for 24 h, and Annexin V-FITC/PI double dye flow cytometry was used to detect the apoptosis of WEHI-231 cells with BP9 treatment. WEHI-231 cells with 0.01 and 10 *μ*g/mL irrelevant peptide treatment were added into 96-well plates as the peptide control, and medium without WEHI-231 cells were added into 96-well plates as the blank control.

### 2.8. Microarray and Data Analysis

The microarray experiment program was conducted by CapitalBio Technology Corporation (Beijing, China) as the previous methods [16]. Simply, WEHI-231 cells were treated with or without 0.01 *μ*g/mL BP9 for 4 h and collected to harvest total RNAs with TRIzol (Invitrogen). The RNA samples were reverse-transcribed and labelled with Cy3 fluorescent dyes, respectively. Then, the labelled cDNA samples were dissolved in 2x GEx Hyb Buffer (HI-RPM) and hybridized onto 8 × 60K Agilent Mouse (V2) Gene Expression Microarray (Agilent Technologies, USA). Subsequently, the hybridized chips were scanned using Agilent Feature Extraction version 10.5.11 software (Agilent Technologies) to extract the signal intensities, which were further analyzed with Agilent GeneSpring GX software. Differentially expressed genes were selected based on the following cut-off criteria. A fold change of 2.0 with a 95% significance level was selected as the threshold for the comparison between both paired cell lines.

### 2.9. Gene Expression Validation

The total RNAs from WEHI-231 cells treated with or without 0.01 *μ*g/mL BP9 was analyzed with the One-Step SYBR® PrimeScript™ RT-PCR Kit (Takara), following the instructions. The differentially expressed genes Sos1, Atg14, Atg12, Csf1, Mlst8, Rragc, and Ube2b were selected to be validated with the specific primers, and *β*-actin was used as housekeeping gene. The primers of selected genes for qRT-PCR were shown in Table S1.

### 2.10. Transmission Electron Microscopy

The autophagy of WEHI-231 cells was evaluated by autophagosome formation screening with a H7650 transmission electron microscope (Hitachi, Japan). WEHI-231 cells were seeded in a six-well plate, treated with 1 and 10 *μ*g/mL BP9 for 24 h, and then collected by centrifugation at 1000× rpm for 10 min. The irrelevant peptide RMYEE 10 *μ*g/mL were used as peptide controls. After twice washing with precooling PBS buffer, 2.5% glutaraldehyde was added to fix WEHI-231 cells for 2 h and then 1% osmium tetroxide was added to WEHI-231 cells for 30 min at 4°C. After dehydration with 50–100% (with 10% gradient) series of ethanol and pure acetone, WEHI-231 cells were embedded with Epon812 resin, cut into thin sections, and stained with uranyl acetate and lead citrate Zi to image the autophagosomes in WEHI-231 cells with the transmission electron microscope.

### 2.11. Western Blotting

WEHI-231 cells were seeded into the 12-well plates and treated with BP9 from 0.001 to 50 *μ*g/mL for 24 h, and then the total proteins from WEHI-231 cells were obtained using the cell culture lysis reagent (Promega), following the instructions. Also, WEHI231 cells were treated with 10 *μ*g/mL irrelevant peptide which was used as a control and rapamycin as a positive control. Western blotting was performed as described previously [22], using mouse anti-mouse LC3 (E1A4007-1, EnoGene, China) to detect protein expression of LC3, a key component of autophagosome formation [23]. Also, AMPK (5832T, CST), p-AMPK (2535T, CST), ULK1 (8054T, CST), p-ULK1 (5869T, CST), and BCL-2 (E1A6139, EnoGene, China) were used to detect the expressions of AMPK-ULK1 phosphorylation, a vital pathway to regulate the autophagy [24, 25]. GAPDH was used as internal reference proteins, and the expression of GAPDH proteins was detected with western blotting using rabbit anti-mouse GAPDH (E12-052-1, EnoGene, China).

### 2.12. Statistical Analysis

Data were expressed as the mean ± standard deviation (SD) of independent experiments. All data analyses were conducted with analysis of variance (ANOVA) using SPSS software. A value of ^∗^ *P* < 0.05, and ^∗∗^ *P* < 0.01.

## 3. Results

### 3.1. Isolation and Homology Analysis of New Bursal-Derived Peptide BP9

**Figure 1:**
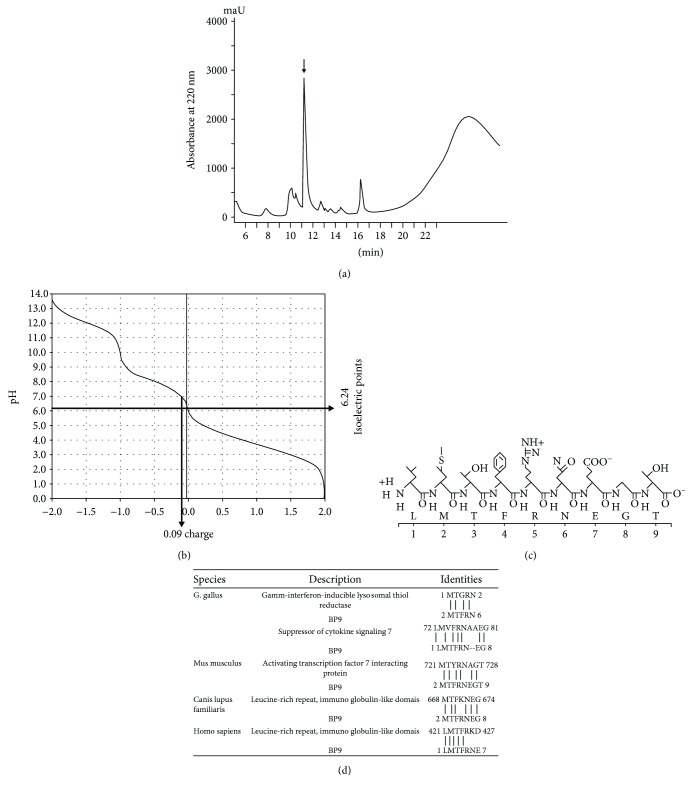
Isolation and identification of BP9. (a) Isolation of BP9. The extract was separated and purified on a 4.6 × 250 mm SinoChrom ODS-BP column with a linear acetonitrile gradient (diagonal line). The retention peak time of BP9 was 11.19 min (the arrow pointed). (b, c) Identification of BP9. Following MALDI-TOF-MS and MS/MS analysis, the amino acid sequence of BP9 was obtained and the chemical formula (b) and the titration curve (c) were analyzed by DNASTAR. The isoelectric point of BP9 was 6.24 with a negative charge of 0.09. (d) The homologous proteins to BP9 in *G. gallus* and other species.

In this paper, following RP-HPLC and MALDI-TOF-MS, we found the strong elution peak at 11.19 min (Figure 1(a)), a new polypeptide isolated from avian BF, whose molecular weight was 1068.22 mz. The further analysis of MS/MS showed that this oligopeptide was consisted of nine amino acids Leu (L), Met (M), Thr (T), Phe (F), Arg (R), Asn (N), Glu (E), Gly (G), and Thr (T) (named after BP9, Figure 1(b)). The chemical formula (Figure 1(b)) and titration curve (Figure 1(c)) of BP9 were analyzed by DNASTAR software, and its isoelectric point of BP9 was 6.24 with a negative charge of 0.09 (Figure 1(c)).

Homology analysis using all nine amino acid sequences LMTFRNEGT as query sequences in the National Center for Biotechnology Information nonredundant and Expressed Sequence Tags databases showed that BP9 has some homology polypeptide sequences with the suppressor of cytokine signaling 7 and gamma-interferon-inducible lysosomal thiol reductase in *G. gallus* (Figure 1(d)). Also, it was found that BP9 was homologous to activating transcription factor 7-interacting protein in *Mus musculus*; leucine-rich repeat, immunoglobulin-like domain, and transmembrane domain-containing protein 3 in *Canis lupus familiaris*; and G protein-coupled receptor 128 in *Homo sapiens* (Figure 1(d)). There results suggested that BP9 might be conserved across species.

### 3.2. BP9 Induced the Strong Antibody Responses Especial to JEV Vaccine in Mouse Immunization Model

**Figure 2:**
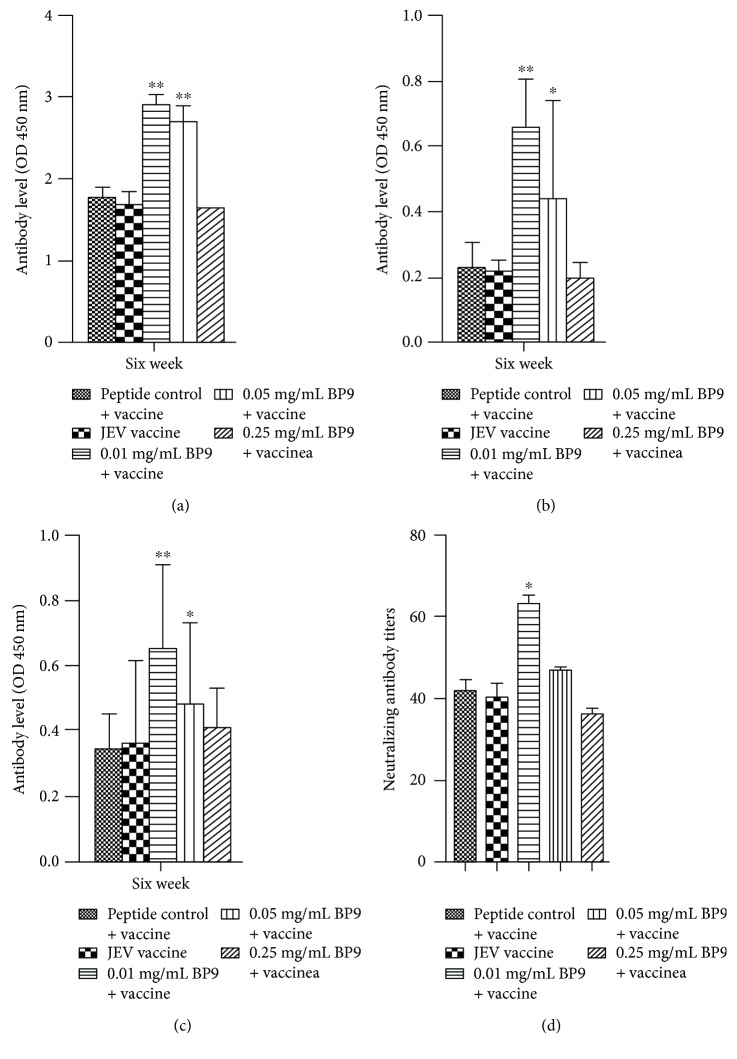
BP9 induced the strong antibody response in JEV vaccine immunization models. Mice were immunized with JEV vaccine and BP9 following prime-boost vaccinations strategy. Total IgG (a), IgG2a (b), and IgG1 (c) were shown. Serum samples from all immunized mice were collected at two, four, and six weeks after the first immunization to detect the IgG and subtype antibody levels by the ELISA method, respectively. (c) Neutralization antibodies. The neutralization antibodies in mice immunized with BP9 and JEV vaccine were measured by plaque formation. Data represent mean + SD. ^∗^ *P* < 0.05 and ^∗∗^ *P* < 0.01, compared with that of the peptide control.

To investigate the inducing effects on antibody production of the new bursal peptide BP9, mice were coimmunized with three dosages BP9 plus JEV vaccine, respectively. The results showed that the antibody productions of mice immunized with the irrelevant peptide plus JEV vaccine (peptide control) was similar to that of JEV vaccine. IgG antibody levels of mice immunized with 0.01 and 0.05 mg/mL BP9 plus JEV vaccine were significantly higher than that of the peptide control (*P* < 0.05), whereas mice immunized with 0.25 mg/mL BP9 plus JEV vaccine produced the similar IgG antibody level to that of the peptide control (Figure 2(a)).

To analyze the antibody subtype especial to JEV, IgG1 and IgG2a antibody levels from all experimental groups were detected with the ELISA method. The results showed that mice immunized with 0.01 and 0.05 mg/mL BP9 plus JEV vaccine produced the significant higher IgG1 antibody levels than that of the peptide control (Figure 2(b); 0.01 mg/mL, *P* < 0.01; 0.05 mg/mL, *P* < 0.05). Also, IgG2a antibody levels produced by mice injected with 0.01 and 0.05 mg/mL BP9 plus JEV vaccine were significantly increased, compared with that of the peptide control (Figure 2(c)).

Furthermore, the results of the neutralizing antibody showed that mice immunized with 0.01 mg/mL BP9 plus JEV vaccine could induce the significant higher neutralizing antibody titers than that of the peptide control, whereas the neutralizing titers of mice immunized with 0.05 and 0.25 mg/mL BP9 plus JEV vaccine were not significantly different to that of the peptide control (Figure 2(d)). These results showed that 0.01 mg/mL BP9 could induce the strong antibody responses in the JEV vaccine immunization model.

Also, three dosages of BP9 without JEV vaccine were injected into mice as the peptide control. It was not found to have the significant humoral immune response to JEV antigen (results not shown), which excluded the miscellaneous effect of polypeptide BP9 on antibody production in the JEV vaccination model.

### 3.3. The Immunomodulatory Functions of BP9 on T Cell Subpopulation and Lymphocyte Viability

To detect the inducing roles of BP9 on cellular-mediated immune responses, in this paper, T cell subtype and lymphocyte viability of all immunized mice were detected with the FCM and MTT method, respectively. The FACS plot results of T cell subpopulations were showed in Figures S1A and S1B and summarized in Figure S1C, in which T cell subtypes of CD3+CD4+ and CD3+CD8+ from mice immunized with 0.01 mg/mL BP9 plus JEV vaccine were similar to that of the peptide control and the CD3+CD4+ and CD3+CD8+ T lymphocyte populations of mice immunized with 0.05 mg/mL BP9 plus JEV vaccine were higher with no significance than that of the peptide control, whereas T cell populations were significantly decreased in mice that injected with 0.25 mg/mL BP9 plus JEV vaccine, compared to that of the peptide control. However, we did not find the significant inducing roles on IL-4 and *γ*IFN cytokine productions in the mice immunized with JEV vaccine and BP9 (results not shown).

Additionally, we found that the splenic lymphocyte viabilities from mice immunized 0.01 and 0.25 mg/mL BP9 plus JEV vaccine were significantly increased, compared to that of the peptide control (Figure S1D). These results suggested that BP9 could modify T cell subtype and stimulate lymphocyte viability in the mouse immunization model.

### 3.4. The Viabilities and Apoptosis of WEHI-231 Cells after BP9 Stimulation

To investigate the role of BP9 on immature B cell viability, in this paper, WEHI-231 cells, a mouse immature B cell model, were treated with BP9 from 0.001 *μ*g/mL to 10 *μ*g/mL for 48 h to detect the proliferation of BP9-treated WEHI-231 cells with the MTT method. The results showed that BP9 could not significantly stimulate WEHI-231 cell viabilities at experimental concentrations (Figure S2A), suggested that BP9 might have not cytotoxic impact on immature B cell viabilities.

Furthermore, the results of FCM with Annexin V-FITC/PI showed that the apoptosis rates of WEHI-231 cells treated with BP9 from 0.001 to 10 *μ*g/mL were decreased, which were lowest with 0.01 and 0.1 *μ*g/mL BP9 among all the experimental groups (Figure S2B). However, the apoptosis rate of WEHI-231 cell with 50 *μ*g/mL BP9 was not significantly different, compared to that of the peptide control. These results indicated that BP9 at low concentration could inhibit the apoptosis of WEHI-231 cells.

### 3.5. Gene Expression Profile Analysis and Evaluation in WEHI-231 Cells with BP9 Treatment

**Figure 3:**
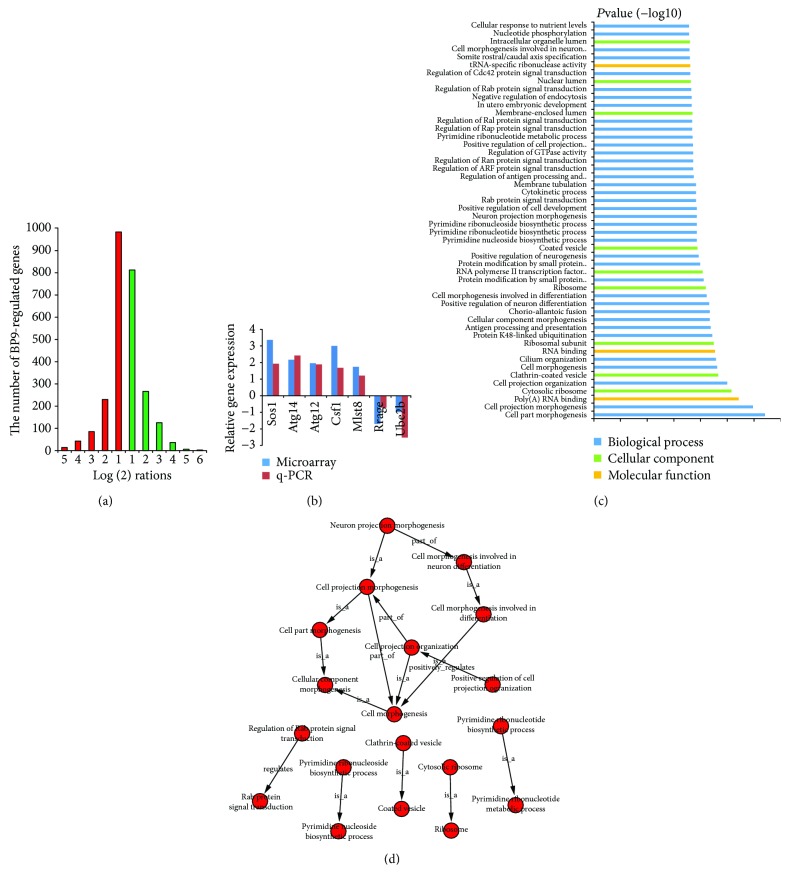
The gene expression profiles and interaction network of top 50 GO terms in preB cells with BP9 treatment. (a) The gene expression profiles of BP9-treated WEHI-231 cells. WEHI-231 cells were treated with 0.01 mg/mL BP9 for 4 h, and the expressions of the differentially regulated genes were detected with microarray; the bar indicated that up- (red) and downregulated (green) genes were higher and lower 2-fold than that of control without BP9 treatment. (b) Gene expression validation. Seven differentially expressed genes obtained from WEHI-231 cells after BP9 treatment were validated with qRT-PCR. (c) The top 50 GO terms selected for finding the vital biological functions in preB cells with BP9 treatment. Blue, green, and orange bars indicated the biological processes, cellular component, and molecular functions among the top 50 GO terms, respectively. (d) Interaction network of top 50 GO terms in preB cells with BP9 treatment. The GO terms were generated by analyzing all the differentially expressed genes in WEHI-231 cells with BP9 treatment.

To explore the mechanism of BP9 on immature B cell differentiation, genome-wide microarray was used to analyze the molecular basis of WEHI-231 cells with 0.01 *μ*g/mL BP9 treatment, which was submitted in GEO with the series record GSE107141. The results of microarray data revealed that 1356 genes were upregulated and 1246 genes were downregulated with 2-fold, compared with that of the control without BP9 treatment, in which the differential expression folds of almost genes were between 1 and −1 log(2) rations (Figure 3(a)).

To independently confirm the results of microarray data, we analyzed Sos1, Atg14, Atg12, Csf1, Mlst8, Rragc, and Ube2b, seven genes involved in immune- and autophagy-related biological processes with qRT-PCR. Results showed that the expressions of five genes Sos1, Atg14, Atg12, Csf1, and Mlst8 were upregulated and Rragc and Ube2b genes were downregulated, which were highly consistent with the results of microarray analysis (Figure 3(b)).

### 3.6. BP9 Induced Various Immune-Related Biological Processes

To further survey the molecular basis of BP9 on the biological functions in immature B cell, the differentially expressed genes in WEHI-231 cells after BP9 treatment were analyzed based on the Gene Ontology (GO) functional annotation, including biological process, cellular component, and molecular function three classifications. We found that among the top 50 significant value of GO terms, 38 terms were belonged to biological process, 9 terms were belonged to cellular component, and the other 3 terms were belonged to molecular function (Figure 3(c)). The net analysis of these significant top 50 terms showed that cell morphogenesis was the major biological processes in WEHI-231 cells with BP9 treatment, which was directly or indirectly related to other various biological processes (Figure 3(d)).

Given the significance of BF on B cell development and antibody production, the immune-related functional processes and genes are the most suitable approach for investigating the mechanism of BP9 on immature B cell differentiation. A summary of the immune-related GO functional annotation was shown in Table 1 and Table S2, including T cell activation, T-helper cell differentiation, MHC molecular, regulation of cytokine secretion. Also, we found that BP9 could significantly regulate various gene expressions involved in autophagy and regulation of autophagy (Table 1).

Interactions among T cell, cytokine, and MHC are the indicative processes during B cell differentiation and development. The extent of overlaps of the differentially regulated genes involved in T cell, cytokine, and MHC processes in BP9-treated WEHI-231 cells was illustrated in Figure S3A. Sequentially, we comprehensively analyzed the interaction net analysis between the differentially expressed genes and the immune-related processes, as shown in Figure S3B, in which among cytokine-related functional processes, the number of the regulated genes involved in negative regulation of cytokine production was mostly in immature B cell after BP9 treatment. Also, we found that the number of cytokine-related functional processes involved by Pycard was more than that of other regulated genes in BP9-treated immature B cells. Furthermore, among T cell-related processes, the number of the regulated genes involved in T cell activation involved in immune response was mostly in WEHI-231 cells after BP9 treatment, and the numbers of T cell-related processes participated by HIX and Irf1 were more than that of other regulated genes. Additionally, upregulated Ifng participated both cytokine- and T cell-related biological processes, suggesting that Ifng might be the important gene in the interaction between T cell and cytokine immune responses. These results suggested that T cell, cytokine, and MHC cellular processes might interact through various gene-interrelated nets during immature B cell differentiation and development.

### 3.7. The Enriched Signal Pathways in Immature B Cell after BP9 Treatment

To investigate the signals activated by BP9 treatment in immature B development, based on known KEGG pathway information, we analyzed the enriched pathways by classification of the differentially regulated genes in WEHI-231 cells after BP9 treatment (Table 2). There were four significantly enriched pathways with *P* values less than 0.05, including ribosome, regulation of autophagy, glycosphingolipid biosynthesis-ganglio series, and RIG-I-like receptor signaling pathway. Additionally, we found that four gene expressions among eight genes involved in the regulation of autophagy were upregulated and four gene expressions were downregulated in WEHI-231 cells with BP9 treatment. Also, five upregulated genes and four downregulated genes were involved in the RIG-I-like receptor signaling pathway in BP9-treated WEHI-231 cells. These results indicated that BP9 might activate various immune-related cellular signals, resulting in B cell development.

### 3.8. BP9 Induced Autophagy and AMPK-ULK1 Phosphorylation in Immature B Cells

**Figure 4:**
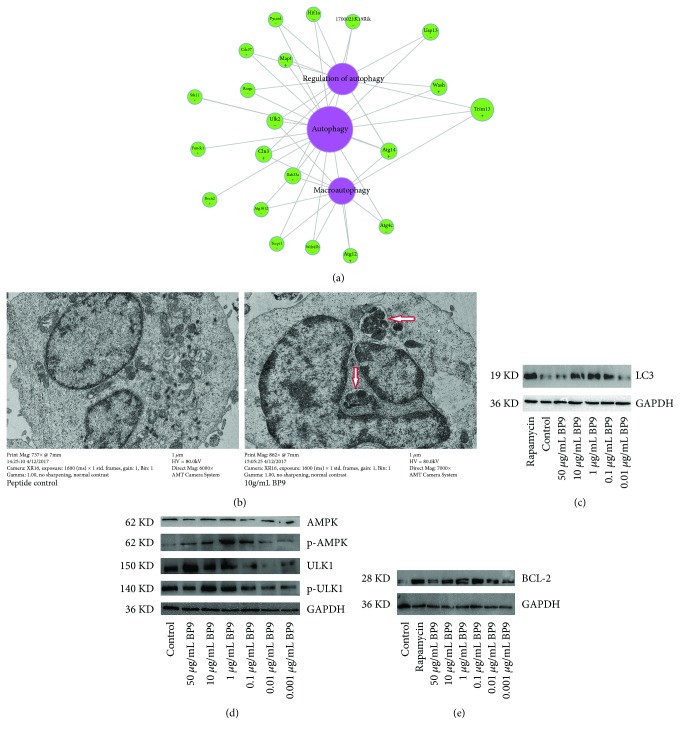
BP9 induced autophagy, enhanced AMPK-ULK1 phosphorylation, and regulated the BCL-2 expression in preB cells. (a) Gene net of the differentially regulated gene involved in autophagy-related biological processes in WEHI-231 immature B cells after BP9 treatment. Purple and green circle colors indicated autophagy-related terms and regulated genes, respectively, and “+” and “−” in green circles indicated up- and downregulated genes, respectively, in BP9-treated preB cells. The bigger the purple circle area size is, the more the number of interacting genes. The bigger the green circle area size is, the more the number of interacting GO terms of these selected immune-related processes in Table S1. (b) Autophagy formation in WEHI-231 cells with 10 *μ*g/mL BP9 treatment following immunofluorescence microscopy. Control with 10 *μ*g/mL irrelevant peptide treatment was used as a control. The red arrow refers to autophagy in WEHI-231 cells with the swollen mitochondria. (c) LC3 protein expression with BP9 treatment in WEHI-231 cells with western blotting analysis. (d) AMPK-ULK1 phosphorylation. WEHI-231 cells were administered after BP9 treatment at experimental concentrations, total proteins were isolated, and the levels of AMPK, phosphorylated AMPK, ULK1, and phosphorylated ULK1 were analyzed by western blot using the indicated antibodies. (e) Expression levels of BCL-2. Similarly, in isolated proteins from BP9-treated WEHI-231 cells, expression levels of BCL-2 protein with BP9 treatment were assessed. In (c), (d), and (e), control with 10 *μ*g/mL irrelevant peptide treatment was used as the irrelevant control (control) and rapamycin as the positive control. Representative western blots are demonstrated.

It was observed that BP9 regulated the differential expressions of 21 genes, which was involved in autophagy, regulation of autophagy, and macroautophagy in the gene network (Figure 4(a)). To detect the role of BP9 on autophagy of WEHI-231 cell, autophagosome formation was observed using a transmission electron microscope and the expression level of autophagy-related gene microtubule-associated protein 1 light chain 3*α* was examined using western blotting analysis. The results of electron microscopy showed that compared with that of the peptide control, we observed the significant autophagy with the swollen mitochondria in WEHI-231 cells with 10 *μ*g/mL BP9 treatment (Figure 4(b)) and found some small autophagosome formation in WEHI-231 cells after 1 *μ*g/mL BP9 treatment (results not shown). Additionally, the expressions of LC3 protein at experimental concentrations from 0.01 to 50 *μ*g/mL were increased in BP9-treated WEHI-231 cells, among which the expression levels of LC3 protein were highest at 10 and 1 *μ*g/mL BP9 treatment (Figure 4(c)). These results proved that BP9 could induce the autophagy in immature B cells, which suggested that autophagy might be an important mechanism during immature B cell differentiation and development.

To investigate the potential signal of BP9 on autophagy in immature B cells, in this paper, we detected the AMPK-ULK1 phosphorylation in WEHI-231 cells with BP9 treatment. Western blotting analysis revealed the dose-dependent increased expressions of p-AMPK by BP9 treatment from 0.1 to 50 *μ*g/mL, in which the expression level of p-AMPK was highest in WEHI-231 cells with 1 *μ*g/mL BP9 treatment. Also, we observed that 50 and 10 *μ*g/mL BP9 induced the increased expressions of ULK1 and 10 and 1 *μ*g/mL BP9 stimulated the increased expressions of p-ULK1 in WEHI-231 cells, compared with that of the peptide control (Figure 4(d)). These results suggested that AMPK-ULK1 phosphorylation might be the activated signal of autophagy in immature B cell with BP9 treatment.

Furthermore, we found that the expressions of Bcl-2 were significantly increased with BP9 treatment at dosages from 0.01 to 10 *μ*g/mL, compared to that of the peptide control (Figure 4(e)), in which 0.1 *μ*g/mL BP9 induced the highest expression of Bcl-2 among these five dosages of BP9 treatment.

## 4. Discussion

BF plays central roles on B cell differentiation and antibody production [3, 4]. However, the activation mechanism of bursal-derived peptide in immature B cell is still to be established.

In this paper, we isolated and identified a new peptide BP9 with nine amino acids from avian BF, which shared the homologous amino acids with the suppressor of cytokine signaling 7 and gamma-interferon-inducible lysosomal thiol reductase in *G. gallus*. The combination of cytokines can induce the activation of the JAK kinase JAK/STAT signaling pathway, in which the suppressor of cytokine signaling is an important regulator [26]. The lysosomal thiol reductase (GILT) induced by interferon gamma is a key component in MHC-II antigen processing and presentation and exhibits thiol reductase activity on IgG substrate [27]. Furthermore, BP9 were conserved in mouse and human species. Therefore, we suspected that BP9 might play certain function on immune induction specific to antigen stimulation.

Mouse immunization experiments were the commonly used animal models to verify the inducing role of active molecules on immune response to the particular antigen immunization [16, 17]. In this paper, it was found that BP9 significantly induced the increased IgG antibody level and neutralizing antibody titers in mice immunized with BP9 plus JEV vaccine in a dose-dependent manner, compared to that of the JEV vaccine control. Although 0.01 and 0.05 mg/mL BP9 plus JEV vaccine could not induce the strong T cell subtype modification, 0.25 mg/mL BP9 plus JEV vaccine significantly decreased the subpopulation of CD3+CD4+ and CD3+CD8+ T cell in the immunized mice. Additionally, BP9 was homologous with the suppressor of cytokine signaling 7 and gamma-interferon-inducible lysosomal thiol reductase. However, it was observed that there were no significant difference on cytokines IL-4 and IFN-*γ* productions between mice immunized with BP9 plus JEV vaccine and JEV vaccine control. In the subsequent work, the mechanism of BP9 on cytokine signal and cytokine activation will be further investigated. These results indicated that BP9 might be a bioactive peptide that could induce the specific antibody responses to vaccine immunization, suggesting an inducing role of BP9 on B cell differentiation during special antigen stimulation.

To identify the molecular basis of BP9 on B cell differentiation, we selected the WEHI-231 cell line as the immature B cell, which is a current cell model to study the function and mechanism of bioactive molecular on B cell signal [28–30]. In the study, following the systematical microarray assay for the functional process analysis, the differentially expressed genes were involved in various cellular process, metabolic process, and biological regulations. Also, cell morphogenesis was the major biological processes with BP9 treatment in WEHI-231 B cell within the network analysis of top 50 GO terms. These results implied that BP9 might activate various cellular biological processes and functions, which were closely related to B cell development.

Interestingly, we found that 34 differentially regulated genes were involved in immune-related functional processes, including T cell, MHC molecular, and cytokine-related biological processes. Furthermore, T cell activation involved in immune response and negative regulation of cytokine production might be both major biological processes in the interaction network of immune-related functional processes and the regulated genes, through which various regulated genes and their involved immune-related processes might interplay and interact in BP9-treated immature B cells. Also, in this paper, we did not observe the significant change in IL-4 and IFN-gamma cytokine production from the immunized mice with BP9 plus JEV vaccine, which might be related to the biological process of negative regulation of cytokine production, in which the mechanism of BP9 on cytokine secretion still be further confirmed.

Additionally, among the differentially regulated genes involved in immune-related GO terms, Hlx and Irf1 were the major genes involved in various T cell-related biological processes, Pycard was one important gene involved in various cytokine-related biological processes, and Ifng was another important gene involved in various T cell and cytokine processes, respectively. Hlx was reported to be expressed at early stages of B-lymphoid differentiation [31]. Interferon regulatory factor 1 (Irf1) was one of key drivers of type 1 regulatory T cell differentiation [32] and participated in counteracting the inhibitory effect of IRF4 on the production of IFN-*γ* [33]. IFN-*γ* is a vital activator and inducer with antiviral, antitumor, and immunoregulatory properties in the immune system [34]. CARD9 is an upstream activator of BCL10 and NF-kappaB signaling, which is important to B cell lymphomas of mucosa-associated lymphoid tissue [35]. The interaction net between the regulated genes and immune-related processes in BP9-treated WEHI-231 cells suggested that various related genes and cellular processes interact and interplay, resulting in B cell development and immune activation response.

Furthermore, the enriched pathways in WEHI-231 cells with BP9 treatment showed the immune-related signals, RIG-I-like receptor signaling pathway. RIG-I-like receptors, the identified pattern recognition receptors (PRRs), were important to initiate antiviral immune responses [36]. Ligand-independent signaling downstream of the Ig*α* cytoplasmic domain drives all bursal stages of B cell development during embryogenesis [37]. These results indicated that the regulated genes involved in signal transduction pathways in WEHI-231 cells with BP9 treatment might be direct or indirect related to the regulation of B cell differentiation. Furthermore, the normal immature B cell was one of common research cell models, which will provide some useful and novelty results in in vivo biology. In the further work, we will launch some research works on the normal B cell to verify the mechanism of BP9 in in vivo biology.

Although there are significant differences between the development and activation of avian and murine B cells, they share some common immunological characteristics and functions, which could provide some vital insight gained with murine studies translated into the avian system. Also, the genomic information of mouse and mouse cells is more detailed than that of chicken species, which is valuable to further explore the signal mechanism of BP9 on B cell development. Furthermore, we will carry out some research on avian B cell lines and chicken to prove the function and mechanism of BP9 on B cell differentiation and antibody development.

Autophagy is an intracellular degradation mechanism, which is involved in the regulation of the adaptive immune response, development of B and T cells, antigen presentation by B cells, and survival of memory lymphocytes and antibody-producing plasma cells [38, 39]. According to the results of high throughput data analysis, we found that BP9 significantly regulated various gene expressions involved in the autophagy pathway and autophagy-related biological processes. The results of transmission electron microscopy proved the increased intracellular autophagosome formation in WEHI-231 cells following BP9 treatment. Also, we found the increased expression of LC3 protein in WEHI-231 cells after BP9 treatment, which was similar to that of the overexpression of LC3A autophagy protein in follicular and diffuse large B cell lymphomas [40]. These results suggested that autophagy might play the roles in immature B cell development and differentiation after BP9 treatment.

It has been reported that autophagy-inducing activity is associated with AMPK phosphorylation [41]. To further investigate the signals of BP9 on autophagy of immature B cell, we detected the expressions of AMPK-ULK1 phosphorylation. The results proved that BP9 could induce the AMPK and ULK1 phosphorylation expression. It was reported that the interaction between AMPK and PS domain of ULK1 was required for ULK1-mediated autophagy [42], which made the phosphorylation of Ser/Thr-rich regions of ULK1 multiple sites, thereby activating ULK1 and directly regulating autophagy [23, 24]. These results suggested that AMPK-ULK1 phosphorylation might be the vital signal to immature B cell autophagy with BP9 stimulation.

Bcl-2 family members were the dual regulators of apoptosis and autophagy [43]. Additionally, we observed that the expression of Bcl2 with 0.1 *μ*g/mL BP9 treatment was higher than that of 10 and 1 *μ*g/mL BP9 treatment. However, we almost did not find the intracellular autophagosome with 0.1 *μ*g/mL BP9 treatment and find multiple swollen mitochondria of autophagy in 10 *μ*g/mL BP9-treated WEHI-231 cells. These results suggested the inhibition of Bcl-2 on BP9-induced autophagy, indicating that the ability of BP9 to induce autophagy in immature B cells was dependent on the experimental concentrations of BP9, in which the mechanism of bursa peptide on B differentiation still need to be further verified.

## 5. Conclusion

In brief, B cell differentiation and development are the complicated biological processes, which involved various pathway processes and signaling activation. In this paper, we identified a new oligopeptide BP9 from avian BF, with the strongly inducing roles on antibody responses specific to JEV vaccine in mouse immunization experiments. Furthermore, BP9 regulated various gene expressions involved in immune-related biological processes and signal pathways and regulated autophagy through AMPK-ULK1 phosphorylation in immature B cells. These results provided the insight for the mechanism of bursal-derived peptide on B cell differentiation and development and offered the important reference for the clinical disease prevention and control and vaccine application in avian species.

## Figures and Tables

**Table 1:** The immune-related GO terms in WEHI-231 cells after BP9 treatment.

Term	ID	Regulated genes	Corrected *P* value
*T cell-related terms*			
Alpha-beta T cell activation involved in immune response	GO:0002287	+5/−2	0.95042
Alpha-beta T cell differentiation involved in immune response	GO:0002293	+5/−2	0.95042
T cell differentiation involved in immune response	GO:0002292	+5/−2	0.95042
CD4-positive, alpha-beta T cell differentiation involved in immune response	GO:0002294	+4/−2	0.95042
T cell activation involved in immune response	GO:0002286	+5/−3	0.95042
Positive regulation of T cell migration	GO:2000406	+3/−1	0.95042
Lymphocyte migration	GO:0072676	+6/−2	0.95042
*T-helper cell-related terms*			
T-helper cell differentiation	GO:0042093	+4/−2	0.95042
T-helper 1 cell differentiation	GO:0045063	+2/−2	0.95042
Negative regulation of type 2 immune response	GO:0002829	+2/−1	0.95042
Negative regulation of T-helper 2 cell differentiation	GO:0045629	+2/−0	0.95042
Positive regulation of T-helper cell differentiation	GO:0045624	+3/−0	0.95042
Positive regulation of T-helper 1 cell differentiation	GO:0045627	+2/−0	0.95042
*MHC molecular related terms*			
Antigen processing and presentation of endogenous peptide antigen via MHC class I	GO:0019885	+1/−1	0.95042
Regulation of MHC class II biosynthetic process	GO:0045346	+1/−2	0.95042
MHC class II biosynthetic process	GO:0045342	+1/−2	0.95042
*Cytokine-related terms*			
Negative regulation of cytokine secretion	GO:0050710	+6/−1	0.95042
Regulation of cytokine secretion	GO:0050707	+9/−4	0.95042
Cytokine secretion	GO:0050663	+10/−4	0.95042
Negative regulation of cytokine secretion involved in immune response	GO:0002740	+1/−1	0.95042
Positive regulation of cytokine secretion	GO:0050715	+5/−4	0.95042
Negative regulation of cytokine production	GO:0001818	+11/−5	0.95042
Positive regulation of interleukin-1 secretion	GO:0050716	+2/−2	0.95042
Positive regulation of interleukin-1 beta secretion	GO:0050718	+2/−2	0.95042
Positive regulation of interleukin-1 production	GO:0032732	+3/−2	0.95042
Regulation of interleukin-1 beta secretion	GO:0050706	+2/−2	0.95042
Regulation of interleukin-1 secretion	GO:0050704	+2/−2	0.95042
Positive regulation of interleukin-1 beta production	GO:0032731	+2/−2	0.95042
Interleukin-1 beta secretion	GO:0050702	+2/−2	0.95042
Interleukin-1 secretion	GO:0050701	+2/−2	0.95042
Negative regulation of interleukin-6 secretion	GO:1900165	+2/−0	0.95042
Interleukin-6 secretion	GO:0072604	+2/−1	0.95042
Negative regulation of type I interferon production	GO:0032480	+1/−2	0.95042
Negative regulation of interferon-beta production	GO:0032688	+1/−1	0.95042
*Autophagy-related terms*			
Autophagy	GO:0006914	+12/−10	0.95042
Regulation of autophagy	GO:0010506	+7/−5	0.95042
Macroautophagy	GO:0016236	+5/−5	0.95042

**Table 2:** The enriched pathways in WEHI-231 cells after BP9 treatment.

# term	ID	Input number	*P* value	Upregulated genes	Downregulated genes
Ribosome	mmu03010	26	0.0000308056538509	Rpl8, Rps27, Rps3a1, Mrps17, Rpl21, Rpsa, Rpl31, Mrps7, Rpl27a, Rpl38, Rps27a, Rpl23a, Rpl31, Gm4925, Rps24, Rps20, Rps26, Rps12, Rpl34, Gm13826, Rpl10a, Rpl23	Rps15a, Rps2, Rpl30, Rpl7a
Regulation of autophagy	mmu04140	8	0.007959	Ifna7, Atg14, Atg12, Ifng	Ulk2, Ifna1, Atg16l2, Atg4c
Glycosphingolipid biosynthesis-ganglio series	mmu00604	4	0.025513	St8sia5, Glb1, St6galnac6	Slc33a1
RIG-I-like receptor signaling pathway	mmu04622	9	0.044117	Ifna7, Mapk14, Atg12, Ifih1, D1Pas1	Dhx58, Mapk12, Ifna1, Map3k7

## Data Availability

The data used and/or analyzed in the current study are available from the corresponding author on reasonable request.

## Abbreviations

BP9: Bursal peptide 9
JEV: Japanese encephalitis virus
AMPK: AMP-activated protein kinase
ULK1: Unc-51-like autophagy-activating kinase 1
BF: The bursa of Fabricius
FBS: Fetal bovine serum
RP-HPLC: Reversed-phase high-performance liquid chromatography
MALDI-TOF-MS: Matrix-assisted laser desorption ionization time-of-flight mass spectrometry
MS/MS: Tandem mass spectrometry
ELISA: Enzyme-linked immunosorbent assay
FCM: Flow cytometry
MTT: 3-(4,5-Dimethylthiazol-2-yl)-2,5-diphenyltetrazolium bromide
CD: Cluster of differentiation
LPS: Lipopolysaccharides
Con A: Concanavalin A
DMSO: Dimethyl sulfoxide
RNA: Ribonucleic acid
cDNA: Complementary DNA
RT-PCR: Real-time polymerase chain reaction
PBS: Phosphate-buffered saline
LC3: Microtubule-associated protein 1A/1B light chain 3
GAPDH: Glyceraldehyde-3-phosphate dehydrogenase
BCL-2: B cell lymphoma 2
SD: Standard deviation
GO: Gene Ontology
KEGG: Kyoto Encyclopedia of Genes and Genomes.

## Supplementary Materials

Supplementary 1 Figure S1: BP9 regulated T cell subtype and lymphocyte viability in the mouse immunization model.

Supplementary 2 Figure S2: viability and apoptosis of WEHI-231 cells with BP9 treatment.

Supplementary 3 Figure S3: the expressions of immune-related GO term genes are altered in immature cells with BP9 treatment.

Supplementary 4 Table S1: qPCR primers of involved genes in BP9-treated WEHI-231 cells.

Supplementary 5 Table S2: the regulated genes involved in immune-related GO terms in BP9-treated WEHI-231 cells.
