# Supplementary figures and images for: The Functions and Mechanism of a New Oligopeptide BP9 from Avian Bursa on Antibody Responses, Immature B Cell, and Autophagy
Source: J Immunol Res. 2019 Jan 6;2019:1574383. doi: 10.1155/2019/1574383 (PMC6339771; doi:10.1155/2019/1574383)

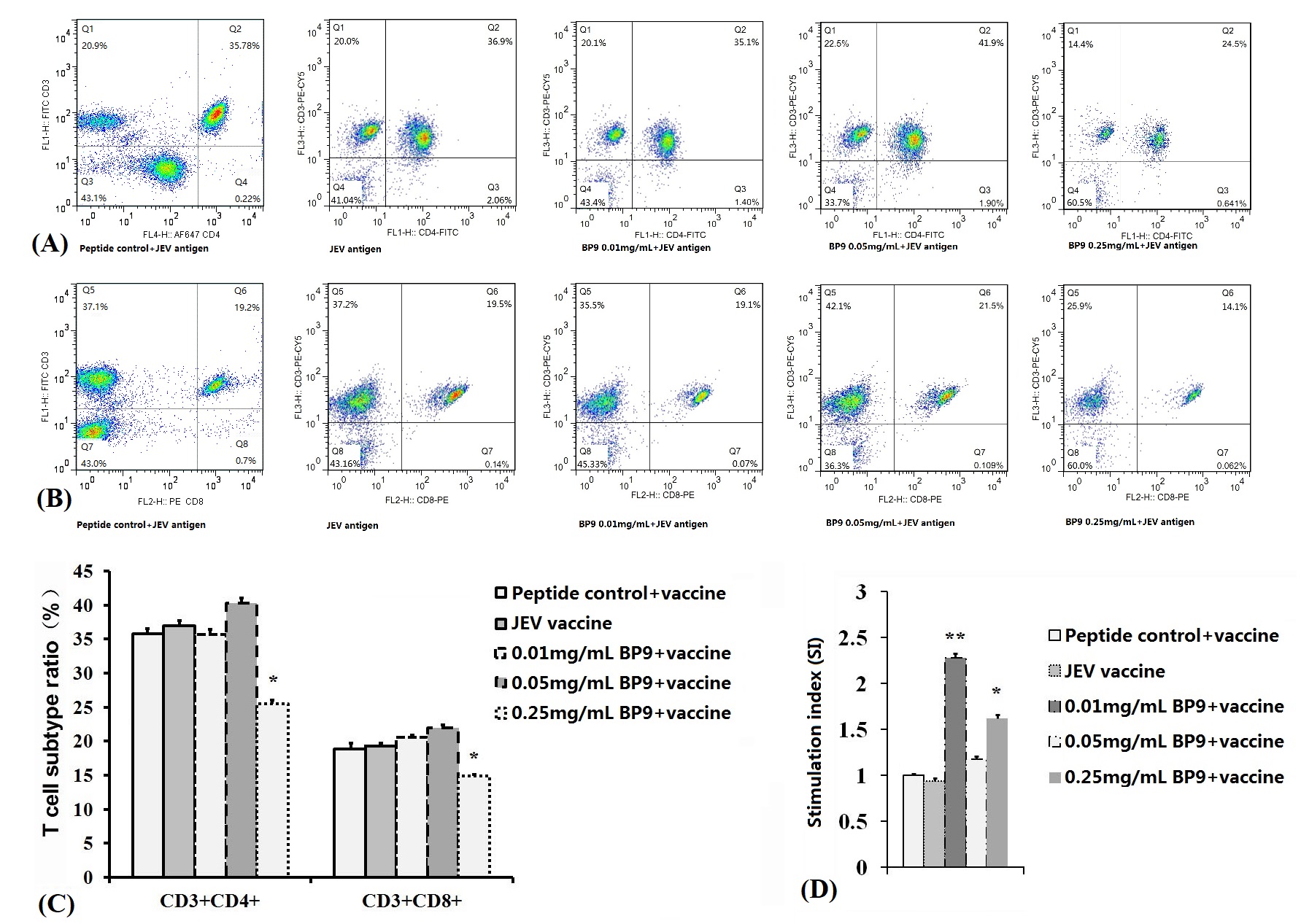

Supplement: Supplementary 1 — Figure S1: BP9 regulated T cell subtype and lymphocyte viability in the mouse immunization model. [file 1574383.f1.jpg]

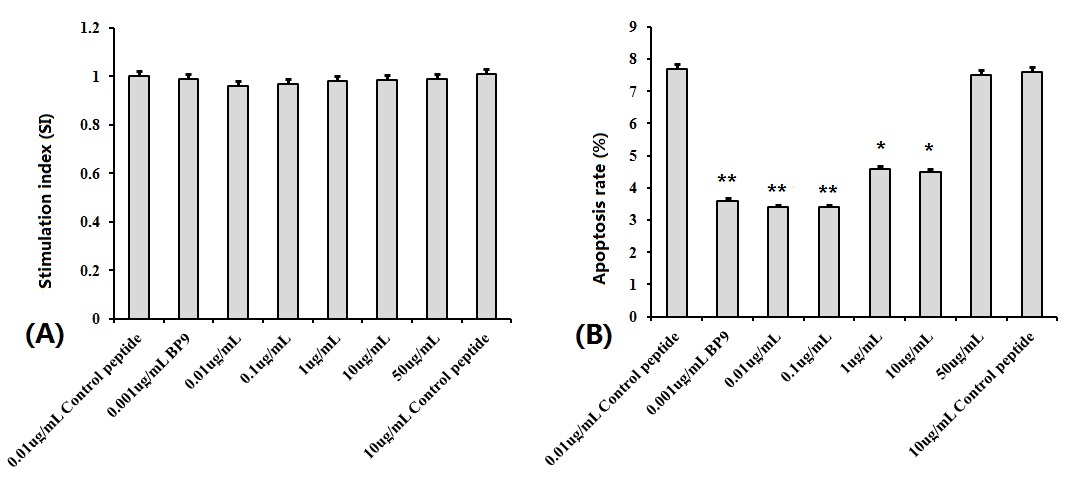

Supplement: Supplementary 2 — Figure S2: viability and apoptosis of WEHI-231 cells with BP9 treatment. [file 1574383.f2.jpg]

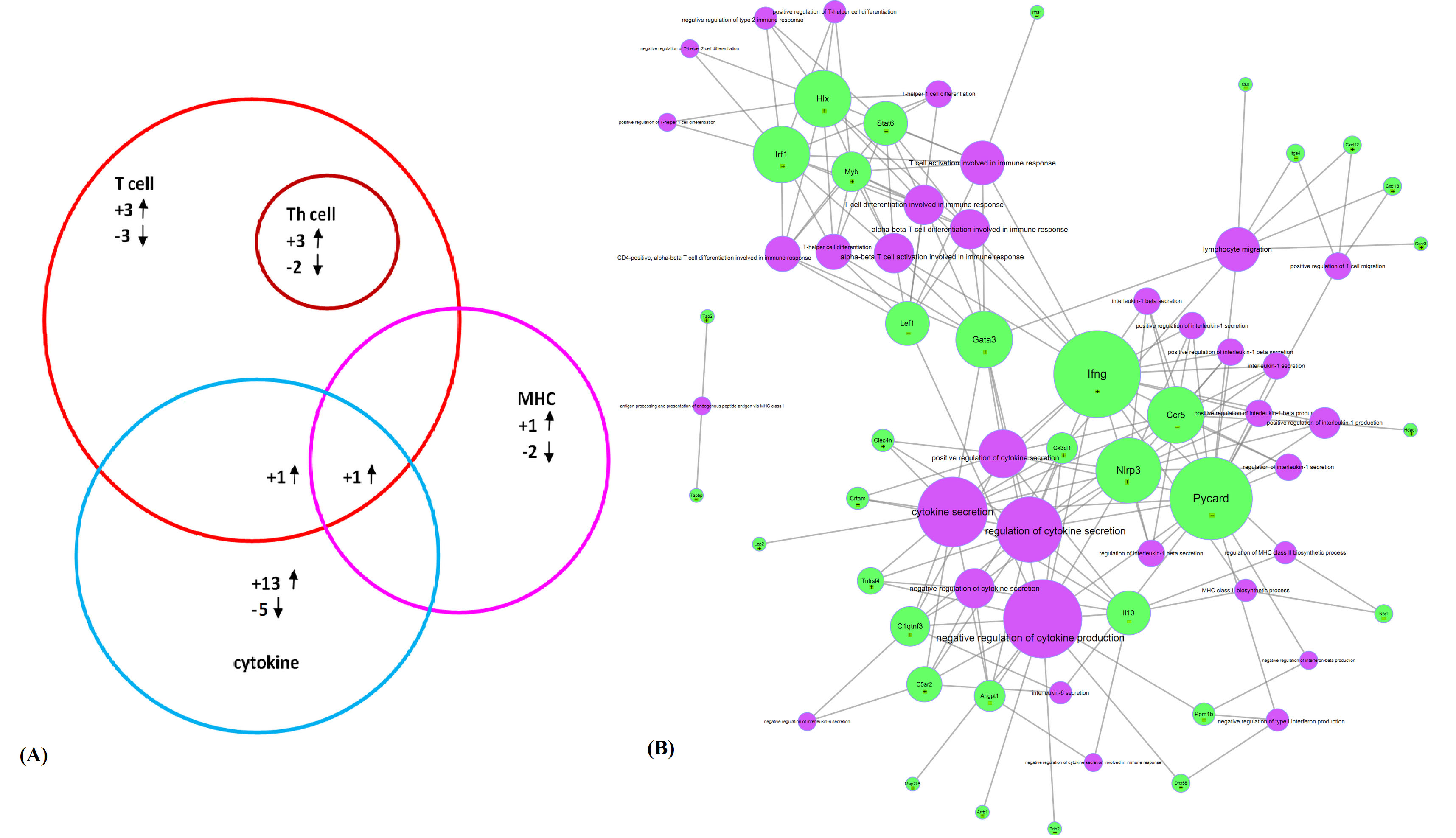

Supplement: Supplementary 3 — Figure S3: the expressions of immune-related GO term genes are altered in immature cells with BP9 treatment. [file 1574383.f3.jpg]
