# Supplementary material for: The Functions and Mechanism of a New Oligopeptide BP9 from Avian Bursa on Antibody Responses, Immature B Cell, and Autophagy
Source: J Immunol Res. 2019 Jan 6;2019:1574383. doi: 10.1155/2019/1574383 (PMC6339771; doi:10.1155/2019/1574383)
Supplement: Supplementary 4 — Table S1: qPCR primers of involved genes in BP9-treated WEHI-231 cells. [file 1574383.f4.doc]

Table S1. qPCR primers of involved genes in BP9-treated WEHI231 cells

| Selected gene | Sequence（from 5’to 3’） |
| --- | --- |
| Sos1 | cacagttgagtggcacataagc |
|  | tttctcaaaccacaaagtgagg |
| Atg14 | atcatattcccaatcgacgaag |
|  | gtggtcatcacagacccatctt |
| Atg12 | cattgtgatccatacctgctgt |
|  | gagaagtgagggttgggagat |
| Csf1 | gagaccctcagacattggattc |
|  | ctggtcagacaacatctggagt |
| Mlst8 | caatagtgccggaaactgctat |
|  | tgtcatcagggagaagttgga |
| Rragc | tccagaaggtggtgtttcataa |
|  | Ttcgtagtcaaaggttggatca |
| Ube2b | cacaccagcctcctagagatgt |
|  | catgcagtaaccactgaaccat |
